# MYOGENIC CONTRIBUTORS TO PHYSICAL FUNCTION IN OLDER ADULTS WITH SYMPTOMATIC KNEE OSTEOARTHRITIS

**DOI:** 10.1093/geroni/igad104.2443

**Published:** 2023-12-21

**Authors:** Alisa Johnson, Jennifer Nichols, Heather Vincent, Roger Fillingim, Yenisel Cruz-Almeida

**Affiliations:** University of Florida, Gainesville, Florida, United States; University of Florida, Gainesville, Florida, United States; University of Florida, Gainesville, Florida, United States; The University of Florida, Gainesville, Florida, United States; The University of Florida, Gainesville, Florida, United States

## Abstract

Knee osteoarthritis (OA) is a leading cause of mobility disability among middle-aged and older adults, and contributes to significant global disease burden. The impact of knee OA on physical function varies widely across individuals, and the factors contributing to this heterogeneity are poorly understood. Recent compelling research suggests that myogenic factors, including muscle quality (MQ), may underly symptoms in knee OA. While MQ is gaining attention in aging research, the role of MQ on functional outcomes in persons with knee OA remains unknown. This pilot study examined morphological and neuromuscular biomarkers of MQ among adults aged 50 years and older (N=44) who met clinical criteria for symptomatic knee OA. Physical performance was assessed using the Short Physical Performance Battery (SPPB). MQ was quantified using B-mode ultrasound (echo intensity) and isokinetic strength measures (force/body mass). Associations between MQ and physical performance were determined, with consideration for clinical pain, age, sex, and body mass index. Pearson correlation analysis indicated SPPB scores were associated with clinical pain, strength, and MQ (r values ranged -.42 – 0.60, p’s< 0.05). Multivariable backward stepwise linear regression analysis demonstrated that MQ biomarkers contributed 39% to variance for SPPB score independent of clinical pain and strength, after adjustment for age, sex, and BMI (b’s = -0.32 – 0.45; p’s < 0.05). MQ is an important consideration for physical function in older adults with chronic knee OA pain, regardless of pain severity, and represents a potentially potent treatment target for disability prevention.

